# Modulation of inter-organ signalling in obese mice by spontaneous physical activity during mammary cancer development

**DOI:** 10.1038/s41598-020-65131-9

**Published:** 2020-05-29

**Authors:** Delphine Le Guennec, Victor Hatte, Marie-Chantal Farges, Stéphanie Rougé, Marie Goepp, Florence Caldefie-Chezet, Marie- Paule Vasson, Adrien Rossary

**Affiliations:** 1 Université Clermont Auvergne, INRAE, UNH, F-63000, Clermont-Ferrand, France; 2 University of Edinburgh Medical School, Centre for Inflammation Research, Queen’s Medical Research Institute, Edinburgh, United Kingdom

**Keywords:** Breast cancer, Risk factors

## Abstract

Accumulative evidence links breast cancer development to excess weight and obesity. During obesity, dysregulations of adipose tissue induce an increase in pro-inflammatory adipokine secretions, such as leptin and oestrogen secretions. Furthermore, a raise in oxidative stress, along with a decrease in antioxidant capacity, induces and maintains chronic inflammation, which creates a permissive environment for cancer development. Physical activity is recommended as a non-pharmacological therapy in both obese and cancer situations. Physical activity is associated with a moderation of acute inflammation, higher antioxidant defences and adipokine regulation, linked to a decrease of tumour-cell proliferation. However, the biological mechanisms underlying the relationship between oxidative stress, low-grade inflammation, carcinogenesis, obesity and physical activity are poorly understood. Our study is based on old, ovariectomised mice (C57BL/6J mice, 33 weeks old), fed with a high fat diet which increases adipose tissue favouring overweight and obesity, and housed in either an enriched environment, promoting physical activity and social interactions, or a standard environment constituting close to sedentary conditions. Our model of mammary carcinogenesis allowed for the exploration of tissue secretions and signalling pathway activation as well as the oxidative status in tumours to clarify the mechanisms involved in a multiple factorial analysis of the data set. The multiple factorial analysis demonstrated that the most important variables linked to moderate, spontaneous physical activity were the increase in growth factor (epithelial growth factor (EGF), hepatocyte growth factor (HGF)) and the activation of the signalling pathways (STAT3, c-jun n-terminal kinases (JNK), EKR1/2, nuclear factor-kappa B (NF-κB)) in the gastrocnemius (G). In inguinal adipose tissue, the NF-κB inflammation pathway was activated, increasing the IL-6 content. The adiponectin plasma (P) level increased and presented an inverse correlation with tumour oxidative status. Altogether, these results demonstrated that spontaneous physical activity in obesity conditions could slow down tumour growth through crosstalk between muscle, adipose tissue and tumour. A spontaneous moderate physical activity was able to modify the inter-organ exchange in a paracrine manner. The different tissues changed their signalling pathways and adipokine/cytokine secretions, such as adiponectin and leptin, resulting in a decrease in anti-oxidative response and inflammation in the tumour environment. This model showed that moderate, spontaneous physical activity suppresses tumour growth via a dialogue between the organs close to the tumour.

## Introduction

Breast cancer is the most common female cancer in the world (25% of female cancers with 1.7 million new diagnoses in 2012) and the second leading cause of death by cancer for women^1^. Some of the strongest associated risk factors for breast cancer development are becoming overweight after menopause and obesity at any age. These factors are also determinants of poor prognosis and high recurrence. In the context of the global epidemic of obesity with a growing prevalence (15,9% in Europe in 2014)^2^, the expansion of adipose tissue as a result of obesity induces an increase in lipolysis, chronic low-grade inflammation and dysregulation of energy homeostasis^3^. However, lipids play a recognized role in tumour growth and development, notably by modulating aromatase activity in the adipose tissue and selective oestrogen receptors in tumours^3^. Moreover, an altered adipokine profile is also observed in excess of weight and adiposity linked to obesity. The secretion of leptin, a pro-inflammatory adipokine, increases differentiation, proliferation and the survival of cancer cells via the Janus Kinase (JAK)/Signal Transducers and Activators of Transcription 3 (STAT3), Mitogen-activated protein kinases (MAPK)/extracellular signal regulated kinases (ERK) and phosphoinositide 3-kinase (PI3K)/protein kinase b (AKT) signalling pathways^4^. On the contrary, adiponectin, a hormone inversely correlated with obesity, is protective against breast cancer through its anti-inflammatory, insulin sensibilization and pro-apoptotic effects^5^. These two hormones, secreted by the adipose tissue (TA), regulate the whole-body metabolism^6^. Physical activity (PA) is recognised as a protective factor against the development of breast cancer, especially in the case of obesity^7^. However, the mechanisms of interaction between physical activity and tumour development are poorly understood.

Cell metabolic dysregulations and reprogramming are hallmarks of cancer cells^8,9^. The uncontrolled cell proliferation of neoplastic cells involves metabolic adjustments such as the Warburg effect. In addition, hypoxia is induced by cell neoplasia faster than neovascularization. This situation modifies the metabolism, creating a symbiosis between hypoxic cells that need lactate and Warburg cells that secrete lactate. This physiological mechanism is derived from muscles. During exercise, lactate accumulates in the cytosol of muscle cells. This lactate can be oxidized in the mitochondria or transported out of the muscle fibres in order to be oxidized in other tissues, such as the myocardium, the muscles oxidative fibres or the liver. The inter-organ exchange of metabolites allows the optimal function of tissues in all conditions^10–12^. Tumour cells and muscle cells have other similarities, linked to hypoxia. During exercise and cancer development, a high production of ROS is observed. Indeed, in cancer cells, an increase in oxidative stress and a diminution of antioxidant capacity induces a chronic inflammatory environment favouring tumour growth^13^. Contrary to cancer cells, in muscle cells, a moderate and transient oxidative stress is observed during exercise, promoting an increase in antioxidant capacity and an acute inflammatory response^14^.

We hypothesise that physical activity, by increasing the needs of muscle cells and by its acute anti-inflammatory response, can induce a competition between metabolites and inflammation status through the inter-organ exchanges with the tumour cells. This hypothesis was sustained by the improvement in the lipid and carbohydrate profiles observed in patients with physical activity therapy^15^. Our purpose was to explore the impact of physical activity on the tissue environment in a model of breast carcinogenesis. We based our study on old and ovariectomised mice (C57BL/6 J mice, 33 weeks old) fed with a high fat diet and housed in either an enriched environment, promoting physical activity and social interactions, or a standard environment constituting close to sedentary condition. The metabolic exchange was analysed between most involved organs around the tumour environment, including healthy mammary gland, inguinal adipose tissue (IAT), gastrocnemius muscle and tumours. Analysed metabolites were adipokines (adiponectin, leptin, resistine), estradiol, interleukin 6 (IL-6), signalling pathways and tumour anti-oxidative response markers. To have a new global approach, a Multi Factorial Analysis (MFA) was conducted on the data set obtained, along with the correlations.

## Materials and methods

### Study design: diet and animals

Female C57BL/6 mice (33 weeks; 29,6 ± 2,2 g) were purchased from the Charles River Laboratory (Lyon, France), housed at 22 °C ± 2 °C in standard laboratory conditions (12-h light and 12-h dark cycle on a reverse light cycle) with *ad libitum* access to diet and water. After 2 weeks of acclimatization, the mice were ovariectomised and randomized into two groups (n = 10), in a standard environment (SE) or in an enriched environment (EE), both groups had a high fat diet until sacrifice. The high-fat diet (4.3 kcal/g) was prepared by SAFE (SAFE, Augy, France) according to the American Institute of Nutrition 93 (AIN-93G) recommendations for laboratory rodent purified diets^16^. Diet composition is detailed in Supplemental Table 1. Enrichment of the environment was obtained by housing the mice in a larger cage (60 * 38 * 20 cm) and providing additional accessories (wheels, nests, tunnels…).

Body weight and spontaneous food intake were measured twice a week throughout the experimental period. Body composition (*n* = 5) in the non-anaesthetized mice was individually measured twice during the experiment (Weeks 5 and 8) by quantitative magnetic resonance imaging (MRI) using an EchoMRI 3-in-1 composition analyser (Echo Medical Systems, Houston, TX). Whole-body energy metabolism and physical activity of a group, which comprises five mice, were measured by indirect calorimetry twice during the experiment, before and after tumour implantation, using a two-cage TSE System PhenoMaster/LabMaster (TSE System, Bad Homburg, Germany)^17^. The mice were placed in calorimetry cages (n = 5 per cage) for 4 days with free access to their diet and water (22 ± 2 °C, 12 h daylight cycle).

The mouse sacrifice day is determined by either a tumour volume (2 cm^3^) limit or a 38-day maximum after implantation. For the control mice, sacrifice occurred randomly during the same time period. The Mice were sacrificed by injection with ketamine/xylazine (i.p., 100/10 mg/kg of body weight) (Sigma Aldrich) and by cardiac puncture. After blood centrifugation (13,800 G for 10 minutes at 4 °C in heparinized tubes), plasma was collected, aliquoted and stored at −80 °C until analysis Several organs were harvested: leg muscle, inguinal adipose tissue, mammary gland (MG) and tumour. The organs were weighed before being frozen at −80 °C until analysis.

### Mammary adenocarcinoma cell line and fat pad implantation

C57BL/6 syngeneic EO771 mammary tumour cells (Center for Stem Cell Research, Houston, TX) is a medullary breast adenocarcinoma cell line isolated from spontaneous tumours in C57BL/6 mice^18^.

The cells were cultured in complete RPMI 1640 medium (Biowest, Nouaille, France) supplemented with 10% fetal calf serum (Biowest), 100 μg/ml of streptomycin (Sigma-Aldrich), 100 U/mL of penicillin (Sigma-Aldrich), 2 mM of glutamine (Sigma-Aldrich) at 37 °C in a humid atmosphere at 5% CO_2_.

Prior to implantation, the cells were detached with trypsin, filtered to prevent cell clumping, added to 100 μl of Matrigel reduced in growth factor (BD Matrigel™ Matrix, BD Biosciences, Bedford, MA) with a density of 5 × 10^5^ cells per 100 μl and kept on ice until administration to mice.

After 5 weeks, mammary neoplastic cells were orthotopically implanted into the fourth right mammary gland using the fat pad technique, with 100 μl of cell suspension (6 mice per group) or with vehicle alone (4 mice per group) (see section below).

Three times a week, the size of the tumours was determined by measuring the perpendicular diameter with a digital calliper. The tumour volume was calculated using the formula V = 4/3π × (width/2)^2^ × (length/2), where width is the smaller of the two measurements.

### Tissue preparation and plasma quantification

#### Tissue preparation

Tumours, inguinal adipose tissue and the right gastrocnemius were cut with a scalpel before being milled with ultra-turrax in an ice bucket. The tissues were sonicated before being frozen at −80 °C for a minimum of 10 minutes. After thawing, the samples were homogenized with ultra-turrax and centrifuged at 500 G for 5 minutes. The supernatant was filtered (40 μm filter) before being aliquoted and then frozen before analysis at −80 °C

Intra-tissue protein quantities and plasma proteins were assayed with a BC kit assays (Interchim, Montluçon, France) based on the Biuret method in multiskan (ThermoFisher Scientific, Villebon sur Yvette, France) at 550 nm.

### Quantification of biomarkers

Total cholesterol, triglycerides and glucose were quantified with commercial kits from ABX Pentra Horiba (Montpellier, France), according to the manufacturer’s instructions.

Plasma levels of 17 β-oestradiol was assayed with the immunoassay EIA kits according to the manufacturer’s instructions (Cayman Chemical, Ann Arbor, MI) with a microplate spectrophotometer reader (Multiskan FC, Thermo Scientific, Waltham, MA).

Using Multiplex Biomarker Immunoassays (cat. MADCYMAG-72K-05) according to the manufacturer’s instructions, metabolic hormones (adiponectin, leptin, resistin, IL-6) were determined in plasma and tissue after appropriate dilution. The mean fluorescence intensity (MFI) was detected by the Multiplex plate reader for all measurements (Luminex System, Bio-Rad Laboratories, Germany) using a Luminex system, Bio-Rad Laboratories software version 4.2.

Using Multiplex Biomarker Immunoassays (cat. kit 48–680MAG and 48–681MA) according to the manufacturer’s instructions, both total and phosphorylated forms of signalling pathways (cAMP response element-binding protein (CREB), JNK, NFκB, p38, ERK1/2, AKT, p70S6K, STAT3 and STAT5) were determined in the tumours after appropriate dilution. The mean fluorescence intensity (MFI) was detected by the Multiplex plate reader for all measurements (Luminex System, Bio-Rad Laboratories, Germany) using a Luminex system, Bio-Rad Laboratories software version 4.2.

### Quantification of oxidative status markers in tumours

#### Determination of total glutathione

Total glutathione (GSH) content was determined by the method of Cereser *et al*.^19^. Briefly, dithiothreitol reduced tumour homogenate for 10 minutes at room temperature, and glutathione ethyl ester was added as an internal standard. After protein precipitation, the supernatant was derivatized by adding ortho-phthalaldehyde (OPA) (Sigma-Aldrich, Saint-Quentin-Fallavier, France). The HPLC separation of GSH–OPA adducts were performed by a UP3 HDO silica-based, reversed-phase C18 column (150 × 3.60 mm, particle size 3 µm) from Phenomenex (Interchim, Montluçon, France) maintained at 37 °C, followed by fluorimetric detection at 420 nm after excitation at 340 nm (Summit HPLC system, Dionex SA, Courtaboeuf, France). Derivatives were eluted using a 10–50% acetonitrile gradient in a 25 mM phosphate buffer at pH 6 for 5 minutes. The flow rate was 0.25 ml/min for an elution run of 20 minutes. Chromatograms were integrated using Chromeleon software from Dionex (Version 6.80, Dionex SA, Courtaboeuf, France). GSH content, calculated using a standard curve plotted under the same conditions, was expressed in µmol/g of protein.

#### Determination of protein thiols

Protein thiols were assayed using the method described by Himmelfarb *et al*.^20^. Free thiol groups oxidized by dithiobis-2-nitrobenzoic acid (Sigma-Aldrich, Saint-Quentin-Fallavier, France) were measured at 405 nm on a microplate spectrophotometer reader, expressed as a ratio to protein content in µmol/g.

#### Determination of lipid hydroperoxides

The amounts of tumour lipid hydroperoxides were determined by the method described by Arab and Steghens^21^. Tissue lysates were treated with buffer reagent (40 mM H_2_SO_4_, 20 mM formic acid, 150 µM iron _D_-gluconate and 120 µM xylenol orange in glycerol) (Sigma-Aldrich, Saint-Quentin-Fallavier, France). A standard curve was obtained using a tert-Butyl hydroperoxide solution. Measurements were made at 570 nm on a microplate spectrophotometer reader. The concentration of lipid hydroperoxides, normalized to the protein content, was in µmol/g.

#### Determination of isoprostanes

The level of tumour isoprostanes was measured with an Elisa kit (No. 516351, Cayman chemical, USA) according to the manufacturer’s instructions and read at 405 nm on a microplate spectrophotometer reader.

#### Glutathione reductase activity

Glutathione reductase (GR) activity was determined as previously described^22^. The tissue lysate was incubated with buffer reagent (100 mM Tris-HCl, 1 mM EDTA, 0.16 mM NADPH and 4.6 mM oxidized glutathione (GSSG), pH 7.4) (Sigma-Aldrich, Saint-Quentin-Fallavier, France). Kinetic NADPH oxidation was followed at 340 nm and 37 °C for 3 minutes in a microplate spectrophotometer reader. GR activity, normalized to the protein content, was in IU/g.

#### Glutathione peroxidase activity

Glutathione peroxidase (GPx) activity resulted in the oxidation of GSH in the presence of tert-Butyl hydroperoxide. Secondarily, GR recycled GSSG in the presence of NADPH^23^. The tissue lysate was incubated with reagents (100 mM Tris-HCl, 1 mM EDTA, 22 mM tert-Butyl hydroperoxide, 5 mM GSH, 0.1 IU/ml GR, 2 mM NADPH, pH 7.4) (Sigma-Aldrich, Saint-Quentin-Fallavier, France). Kinetic NADPH oxidation due to GSH recycling was followed at 340 nm and 37 °C in a microplate spectrophotometer reader. GPx activity, normalized to the protein content, was in IU/g.

#### Glutathione S-transferase activity

Glutathione *S*-transferase (GST) activity was quantified as previously described^22^ using the conjugation reaction of GSH with artificial substrate 1-chloro-2,4-dinitrobenzene. The tissue lysate was incubated with reagents (50 mM HEPES, 5 mM GSH, 1 mM 1-chloro-2,4-dinitrobenzene, pH 7.4) (Sigma-Aldrich, Saint-Quentin-Fallavier, France). The 1-chloro-2,4-dinitrobenzene-glutathionylation kinetics were followed at 340 nm and 37 °C in a microplate spectrophotometer reader. GST activity, normalized to the protein content, was in IU/g.

#### Cyclooxygenase activities

The activity of cyclooxygenase (COX) 2 and 1 were measured with a kit (Cayman Chemical Company, Ann Arbor, USA, No 760151) according to the manufacturer’s instructions. Kinetic measurements were performed at 590 nm and 37 °C in a microplate spectrophotometer reader. COX-2 and 1 activities, normalized to the protein content, were in IU/g.

#### Determination of thioredoxin reductase activity

The activity of the enzyme thioredoxin reductase was analysed in the tumours with a kit CAK1042 (Bioscience Cohesion, London, UK) according to the manufacturer’s instructions. The reading was performed by Multiskan at 405 nm for 5 minutes. Thioredoxin reductase activity, normalised to the protein content, was in UI/g.

#### Heme oxygenase 1 activity

The catalytic activity of heme oxygenase 1 was measured by the mixture of the cofactor NADPH, H + (2 mg/mL) (Sigma Aldrich) with ferric heme in a Tris buffer solution (pH 7.4, Tris-Hcl at 100 mM and MgCl_2_ at 2 mM) (Tris: Biosolve, Dieuze, France; MgCl_2_: Prolabo, Nantes, France). The mixture was read by spectrophotometry at 405 nm.

### Data analyses

The first part of the statistical analyses were calculated using R, version 3.2.2. 5,93% of the data were missing. This problem was handled by imputation of the missing values using Expectation-Maximization algorithm^24^ implemented in the MissMDA R package. Data were centered and scaled.

A Multi-Factor Analysis^25,26^ was performed using the R package FactomineR as described in^27,28^. Eight groups were identified: muscle masses, adipose tissue masses, tumour growth, tumour oxidative status, tumour biology, inguinal adipose tissue biology, right gastrocnemius biology and plasma biology (composition of groups of variables in supplemental Table 2). Correlations were calculated using the corrplot R package.

In order to obtain the most relevant metabolites from the first statistical analysis, a second statistical analysis was performed. The second phase of statistical analyses was performed with GraphPad Prism 5 software (GraphPad Software, Inc., San Diego, CA). Pairwise comparisons were calculated with a Mann Whitney t test. Comparisons of three or more groups were made with a 1-way ANOVA when only one parameter was changed or with a 2-way ANOVA when 2 factors were modified. These comparisons were followed by a Bonferroni post test. Statistical means are p ≤ 0.05 (*), p < 0.001 (**) and p < 0.0001 (***). All data were presented as mean ± SEM.

### Ethics approval

This study was approved by the Animal Experimental Committee (Comité Régional d’Ethique sur l’Expérimentation Animale, No. 01095.02, Clermont-Ferrand, France) and carried out in accordance with the ethical guidelines.

## Results

### Body composition, physical activity and mammary tumour growth

All of the mice used in the study were fed with a high-fat diet. Only the housing environment was different with a standard environment (SE) and an enriched environment (EE), the latter favouring physical activity (PA) and social interactions.

Respiratory quotient was similar between the groups. Food intake and energy expenditure were also the same regardless of the environment (data not shown). Despite PA, no changes were observed in the body weights or body composition of the mice. In both groups, weight increased throughout the experiment, mostly due to the high-fat diet (data not shown), without differences in lean mass, adipose mass (data not shown) or distribution.

**Figure 1:**
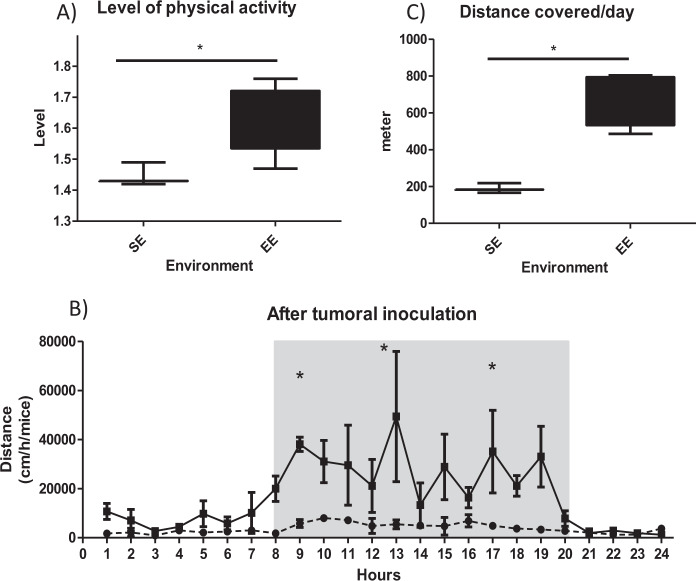
Physical activity in SE and EE. (**A**) The level of physical activity. (**A**) corresponds to total energy expenditure divided by the minimum energy expenditure, for a mouse. Results are mean ± SEM (*n* = 5/group). Data were analysed by Mann Whitney t-test. Representative data are shown: *p < 0.05, SE *vs*. EE. (**B**) Physical activity recording over a 24 h period. Results, expressed in mean distance covered per hour and per mouse, are mean ± SEM (*n* = 5/group). Data were analysed by ANOVA repeated measures. Representative data are shown: *p < 0.05, SE *vs*. EE. (**C**) Total distance per mouse per day. Results (meters) are mean ± SEM (*n* = 5/group). Data were analysed by Mann Whitney t-test. Representative data are shown: *p < 0.05, SE *vs*. EE.

As expected, PA was different between the two groups. The level of physical activity, represented by the total energy expenditure divided by the minimum energy expenditure, was higher in the EE than in the SE (Fig. 1A). Physical activity recording over a 24 h period revealed an increase in mean distance covered per hour and per mouse in the EE group (Fig. 1B), resulting in a higher total distance covered per mouse in the EE than in the SE (Fig. 1C).

**Figure 2:**
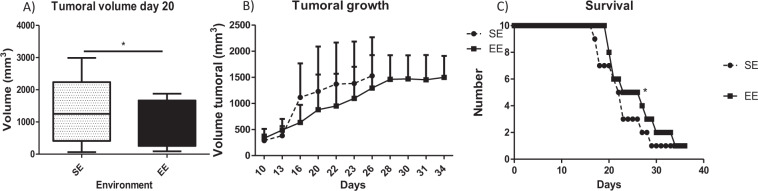
Tumour development in SE and EE. (**A**): Tumour volume at day 20 in mm^3^ depending on the environment. Results are mean ± SEM (*n* = 10/group). Data were analysed by Mann Whitney t-test. Representative data are shown: *p <0.05, SE *vs*. EE. (**B**) Time course of tumour volume to reach 2000 mm^3^. Results are mean ± SEM (*n* = 10/group). Data were analysed by ANOVA repeated measures. Representative data are shown: *p < 0.05, **p < 0.01, SE *vs*. EE. (**C**) Time course survival in terms of end-point to sacrifice. Results are mean ± SEM (*n* = 10/group). Data were analysed by Mantel-Cox . Representative data are shown: *p < 0.05, SE *vs*. EE.

After EO771 cancer cell implantation, tumour growth was significantly lower for the mice housed in the EE *vs*. the SE (Fig. 2A). Mammary tumour growth took longer to reach 2 cm^3^ for the group housed in the EE than in the SE (Fig. 2B), resulting in a better survival rate for the mice in the EE group than in the SE, with respect to the experimental end-points (Fig. 2C).

### Multiple factorial analyses

No differences were perceived in terms of anatomy or weight between the groups housed in standard or enriched environments. However, even in the case of obesity, the enriched environment slowed down tumour growth. This is why we have focused on the biological effects, especially the inter-organ exchange. A particular interest was placed on tissue implied in physical activity, adiposity and mammary carcinoma (i.e. gastrocnemius, inguinal adipose tissue, plasma and tumour). In view of the relatively large amount of variables, a MFA was performed in order to have a comprehensive and global approach to the effects of moderate physical activity on mammary cancer development. Data were sorted into height groups of quantitative variables and one qualitative variable corresponding to the environment to improve readability without losing information. The MFA defined 11 principal components, called dimension (Dim), to explain the data set variability. The qualitative variable “environment” showed a high contributive value in each of the first two Dims, which explained 34,4% of the total variance.

**Figure 3:**
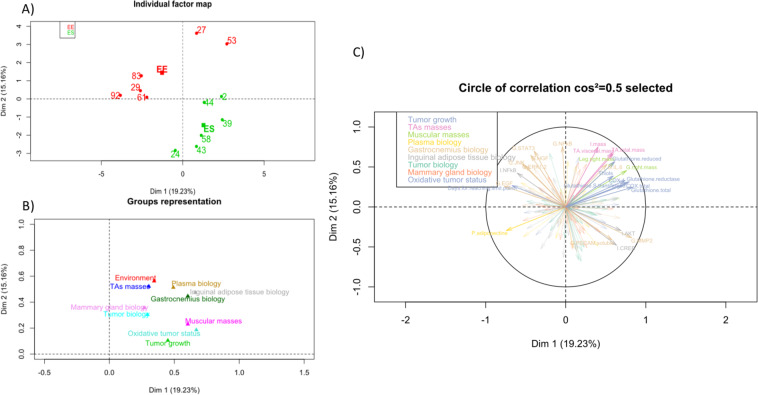
Multiple factorial Analyses – Contribution of the variables in the two dimensions. (**A**) Individual plot according to the first 2 dimensions. Group in green is the SE and group in red is the EE. (**B**) Variable groups plot according to the first 2 dimensions. (**C**) Most important variables in dimensions 1 and 2. Only the variables having a cos2 greater than 0.5 were shown.

This association of Dim1 and Dim2 discriminated, in the individual plot, the mice into two distinct groups according to their housing environment (Fig. 3A). In the first two Dims, among the variable groups, the main contributors were the biological parameters for plasma, gastrocnemius and inguinal adipose tissue (Fig. 3B), for which enriched environment (EE) presented a significant impact. Moreover, Dim1 was mainly represented by the muscular mass and the tumour growth (Fig. 3B and Table 1) whereas Dim2 was more affected by the adipose tissue masses and the environment (Fig. 3B and Table 2). Tables 1 and 2 resume the significant contributive variables inside each variable group for the two Dims in terms of correlation factor and *p*-value. To investigate additional differences between the mouse groups and the Dim signification, the MFA parameters representing each variable in our multi-dimensional space (cos2) were also plotted against the two Dims (Fig. 3C). For more clarity, only the variables with cos2 > 0.5 were represented on Fig. 3C.

**Table 1:** Contributions of variables to dimension 1.

Groups	*Variables*	Correlations	P-value
Environment	Environment	0.3448	0.0447
	ES	1.2721	0.0447
	EE	−1.2721	0.0447
Tumour growth	Days to reach limit point	−0.6698	0.0172
Tumour oxidative status	Glutathione total	0.8352	7.00E-04
	Glutathione reductase	0.7932	0.0021
	COX total	0.7584	0.0043
	COX-1	0.7401	0.0059
	Glutathione-S-transferase	0.7074	0.0101
	Heme oxygenase	0.6612	0.0192
	Glutathione reduced	0.6119	0.0345
	Thiols	0.5927	0.0423
Tumour biology	ERK1/2	0.6171	0.0325
Adipose tissue masses	visceral mass	0.5937	0.0418
	total mass	0.5811	0.0475
Inguinal adipose tissue biology	AKT	0.6905	0.0129
	CREB	0.6342	0.0268
	p38 MAPK	0.6555	0.0206
	TNF-α	−0.5852	0.0456
	NFkB	−0.6071	0.0363
Plasma biology	Adiponectin	−0.7437	0.0056
Gastrocnemius biology	MMP2	0.8228	0.001
	Leptin	0.6761	0.0158
	PECAM soluble	0.5787	0.0487
	EGF	−0.7671	0.0036
Muscles masses	Right G mass	0.7594	0.0042
	Left leg mass	0.6704	0.017
	Right leg mass	0.5998	0.0393

Results are mean ± SEM (*n* = 6/group). Data were analysed by multiple factorial analysis.

**Table 2:** Contributions of variables to dimension 2.

Groups	*Variables*	Correlations	P-value
Environment	Environment	0.5628	0.0049
	EE	1.4431	0.0049
	ES	−1.4431	0.0049
Tumour oxidative status	Isoprostanes	0.6168	0.0326
Tumour biology	EGF	0.6665	0.0179
	MMP2	−0.6606	0.0194
	MMP3	−0.679	0.0152
Adipose tissue masses	Inguinal mass	0.7506	0.0049
	total mass	0.6781	0.0154
	Visceral mass	0.6653	0.0182
Inguinal adipose tissue biology	MMP2	−0.6283	0.0287
Gastrocnemius biology	NFκB	0.7554	0.0045
	STAT3	0.6983	0.0115
	HGF	0.6455	0.0234

Results are mean ± SEM (*n* = 6/group). Data were analysed by multiple factorial analysis.

The qualitative variable “environment enriched” was associated negatively with Dim1 (r² = −1.2721, p = 0.0447) and positively with Dim2 (r² = 1.4431, p = 0.0049). Concerning Dim1, among the 25 variables associated, the most important variables and the main positive contributors were variables of the tumour oxidative status with 8 variables (total glutathione (r² = 0.8352, p = 7.00E-04), total COX activity (r² = 0.7584, p = 0.0043), heme oxygenase activity (r² = 0.6612, p = 0.0192) and thiols (r² = 0.5927, p = 0.0423). The gastrocnemius was also highly positive contributor in Dim1 with 6 variables mainly represented by the gastrocnemius biology (leptin (r² = 0.6761, p = 0.0158), PECAM soluble (r² = 0.5787, p = 0.0487)) and the muscle masses. The last positive contributors were the inguinal adipose tissue biology, notably, the activated signalling pathway of IAT (AKT (r² = 0.6905, p = 0.0129), p38 (r² = 0.6555, p = 0.0206), CREB (r² = 0.6342, p = 0.0268)). Five of the 25 variables were negatively associated in Dim1 (days to reach limit point, gastrocnemius EGF, IAT NFκB and TNF-α). The plasma amount of adiponectin was the last negatively associated variable in Dim1 (r² = −0.7437, p = 0.0056) and the only plasma variable involved in the definition of these Dims. Concerning Dim2, only 11 variables were implicated. As for Dim1, adipose tissue masses (total adipose tissues (r² = 0.6781, p = 0.0154), IAT (r² = 0.7506, p = 0.0049), visceral adipose tissue (r² = 0.6653, p = 0.0182)) and the gastrocnemius signalling pathway (NFκB (r² = 0.7554, p = 0.0045) and STAT3 (r² = 0.6983, p = 0.0115)) were major positive contributors. Tumour oxidative status was represented by the isoprostanes levels (r² = 0.6168, p = 0.0326). Three variables were negatively associated with this Dim and reflected tissue remodelling (IAT MMP2 (r² = −0.6283, p = 0.0287), tumour MMP2 and MMP3 (r² = 60.6606, p = 0.0194; r² = −0.6790, p = 0.0152 respectively)).

To conclude, MFA demonstrated that the most important variables for the impact of spontaneous physical activity during breast cancer development in situations of obesity were the adipose tissue mass linked to the circulating adiponectin level, the tumour oxidative status and the tissue signalling pathways. These variables could explained the differences observed between the mouse groups and highlight the metabolic pathways implied.

### Correlation matrix

**Figure 4:**
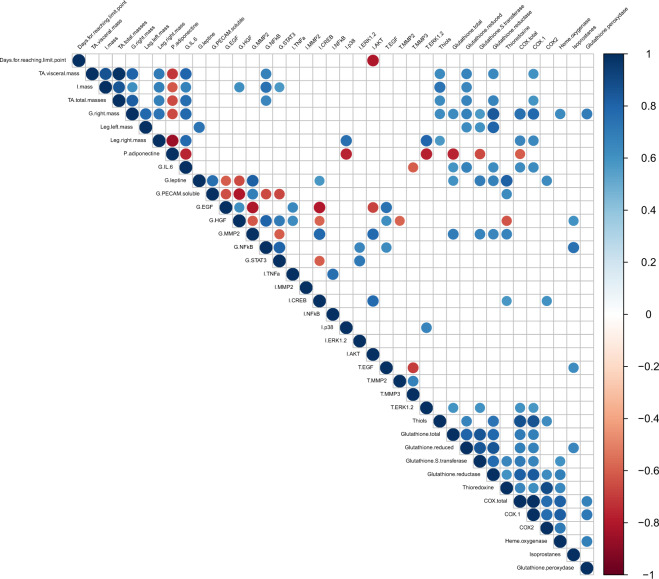
Correlation matrix. Correlations between significant variables contributing to Dim1 and 2. Only significant correlations with a p-value of less than 0.05 were shown.

For more clarity, the correlation matrix was performed on the significant MFA variables and only the significant correlations (p-value ≤ 0.05) were represented (Fig. 4). The correlation matrix plot was insightful in highlighting the relations between the array of variables considered in our model.

Among the plasma parameters only adiponectin emerged from the MFA. Plasma adiponectin had a significant inverse correlation with all of the adipose tissue masses (i.e. total, visceral and inguinal). The amount of plasma adiponectin correlated inversely with the activation of proliferation signalling pathways such as tumour ERK1/2 and IAT p38. Circulating adiponectin presented an inverse correlation with gastrocnemius IL-6 and several tumour antioxidant factors (total glutathione, glutathione S-transferase and total COXs activities).

All the adipose tissue masses had a positive correlation with each other. The different masses of adipose tissue were positively correlated with different inflammation and oxidative markers: the activation of NFκB in the gastrocnemius; the amount of thiol, reduced glutathione, glutathione reductase activity and COX-1 in the tumour.

The mass of the right gastrocnemius was positively correlated: firstly, with the masses of adipose tissue (total, inguinal and visceral) and with the masses of the leg muscles (right and left); secondly, with the production of IL-6 in the right gastrocnemius; thirdly, with different tumour oxidative protection markers (protein thiols, total and reduced glutathione amount, glutathione S-transferase and glutathione reductase activity, heme oxygenase and glutathione peroxidase activity) as well as two oxidative stress markers which were total COXs and COX-1. Finally, the mass of the right gastrocnemius was inversely correlated with the amount of plasma adiponectin.

MFA analyses highlighted the importance of the secreted parameters (myo-adipo-cytokines and growth factors) from the right gastrocnemius in conjunction with the environment. Firstly, the amount of IL-6 in the gastrocnemius was positively correlated with leg masses but also fat tissue masses (total, inguinal and visceral). An inverse correlation was found between the amount of muscle IL-6, the amount of circulating adiponectin and the amount of tumour MMP3. Gastrocnemius IL-6 was also positively associated with different markers of tumour oxidative response (total and reduced glutathione, enzymatic activity of glutathione reductase, COXs total and COX-1). Secondly, the amount of leptin in the gastrocnemius correlated positively with the amount of soluble PECAM and MMP2 in the same tissue as well as with different actors of the tumour oxidative status (total glutathione, glutathione-S-transferase, glutathione reductase, thioredoxin and COX-2). An inverse correlation was found between the amount of leptin and the amount of EGF and of HGF in the gastrocnemius. Thirdly, the growth factors EGF and HGF in the gastrocnemius have the same correlation profile: positive between them and with the activation of the NFκB inflammation pathway in inguinal adipose tissue, and also with the amount of tumour EGF. Inverse correlations were found with the amount of MMP2 in gastrocnemius and the activation of the CREB pathway in inguinal adipose tissue. Lastly, gastrocnemius sPECAM appeared to have mainly local correlations. Positive correlations were observed with leptin and MMP2. On the other hand, negative correlations were observed with growth factors (EGF and HGF) and the activation of signalling pathways (NFκB and STAT3).

Among the tumour oxidative status, all the effectors of the antioxidant response correlated positively together, excepted the amount of tumour isoprostanes. The amount of tumour isoprostanes had a singular behaviour and uncorrelated with tumour oxidative status (except for reduced glutathione). Tumour isoprostanes were positively associated with EGF in tumours and with HGF and NFκB activation in the gastrocnemius.

## Discussion

The aim of this study was to investigate the impact of spontaneous, moderate physical activity on the development of mammary cancer in old, ovariectomised and obese mice. Mice, fed with the same high fat diet, were then divided into two groups, one housed in a standard environment and the other in an enriched environment.

As expected, the mice housed in an enriched environment had a greater physical activity without change in energy expenditure, body composition or circulating metabolites such as glucose, triglycerides or cholesterol. This lack of change can be explained by the short time frame of the experience, there being no more than 12 weeks until the sacrifice. It is currently recognised in the literature, which mainly focuses on high intensive training, that sequential intensive training over a period of more than 12 weeks has a positive impact in diet-induced obesity models^29^. In such conditions, physical activity has demonstrated the ability to correct biological and body parameters in obese mice^29^. In our conditions, despite an adiposity increase of around 20% of body composition, the biological disorders related to metabolic syndrome only just have time to appear. Further, the physical activity in our study is not imposed but spontaneous. In terms of intensity, our physical activity intervention improved regular movement throughout the day, which only corresponds to moderate intensity. In this regard, we did not expect an impact of the spontaneous moderate physical activity on these parameters in our model as it is less stressful and intensive. Yet, our physical activity intervention did, in fact, enable a change in tumour growth. It is for this reason that we decided to focus on the inter-organ dialogue around the tumour environment.

To highlight the effects of physical exercise, we choose to focus on the right gastrocnemius. This muscle is the largest of the paw and has glycolytic and hybrid mixt myosin^30^. Moreover, the gastrocnemius manifests an important response to physical activity notably myokine secretions and biological effects. Finally, the right gastrocnemius is close to the tumour and could impact the tumour environment. In parallel, the impact of adipose tissues on carcinogenesis and on the whole-body homeostasis was evaluated principally by the biological parameters of the inguinal adipose tissue in our study. This tissue is the closest fat deposition to the tumour and appears to be a part of the tumour environment crosstalk^31,32^. Lastly, the left fourth mammary gland of the mouse was considered in order to measure the impact of our intervention on healthy tissue. The inter-organ crosstalk between adipose tissue, muscle, mammary tumour and plasma was investigated in terms of inflammation, oxidative stress, tissue secretions and energy metabolites.

Among the 133 variables studied in our model, a few of them are statistically related to the moderate physical activity ensured by the enriched environment. It is interesting to note that most of the variables associated with the enriched environment are related to cytokine and hormonal crosstalk. Surprisingly, the main cytokines, IL-6 and TNF-α, usually assayed in cancer models, presented no changes in our conditions^13^. Conversely, the two major adipokines, adiponectin and leptin, as well as some growth factors (EGF, VEGF-α and HGF) seemed to play a major role in the tissue crosstalk around the tumour. Other factors that plays a role in tissue remodelling and angiogenesis (MMPs), and usually observed in cancer models^9^, also presented changes in our study.

It appears that moderate physical activity could change inflammation and hormonal parameters both at the systemic and tissue levels^7,33^. Physical activity is recognized as being able to modify adipose tissue secretions notably in a situation of obesity in animal models and clinical trials^15,33,34^. Adipose tissue have a major role in inflammation both at systemic and tissue levels in various chronic diseases (i.e. cardiovascular, metabolic, cancer) that is why the amount of fat tissue is an important factor in chronic inflammation, particularly when due to obesity^3,34,35^. Our results showed that despite regular physical activity, the amount of adipose tissue plays a very strong role in tumour development, particularly through adipokine production (adiponectin, leptin). Interestingly, in our model, a localized inflammation is presented in the inguinal adipose tissue. The amount of TNF-α is linked to an increase in the activation of the NFκB signalling pathway in this tissue. Conversely, a decrease in activation is observed in the IAT signalling pathways AKT, CREB, p38. In the literature, obesity promotes CREB activation resulting in a decrease in adiponectin secretion^36^. This previous observation is in agreement with the increase in the amount of adiponectin in the inguinal adipose tissue and in the plasma observed in our study in response to the decrease of CREB activation. In our model, these results suggest that a part of the anti-inflammatory and the anti-carcinogenesis effects of physical activity is supported by the anti-inflammatory effects of adiponectin. This observation is reinforced by the inverse correlations between circulating adiponectin and the various tumour antioxidant markers. What is more, another adipokine is modified by physical activity leptin in our model. The gastrocnemius and tumour leptin levels decrease without changes in IAT or plasma levels. According to the literature, physical activity induces a more important leptin captation by muscular cells^37^. Regular exercise decreases plasma levels of leptin and improves insulin sensitivity, mainly due to the leptin receptors in muscle cells^38^. In this context of muscle exercise, leptin signalling will increase the signal to insulin and trigger a better uptake of the glucose required for physical activity^37^. In parallel, leptin, an important pro-carcinogenesis adipokine, is strongly captured by tumour cells because it promotes cell proliferation by activating main signalling pathways such as JNK, AKT, p38^5^. We could hypothesise that a competition between muscles and tumour appears. Leptin is not available for tumour growth while physical activity consumes it. This is highlighted by the gastrocnemius leptin decrease and the tumour oxidative and inflammatory status changes.

Physical activity also induces changes in muscle. Notably, the STAT3 cell-signalling pathway, particularly responsive to leptin, is activated to a greater extent. This result confirms the strong and increased consumption of leptin in the gastrocnemius due to physical activity. In parallel, the inflammation-signalling pathway, NFκB, is activated more in the gastrocnemius of the mice housed in the enriched environment. This result is in agreement with the literature, which describes an acute and local inflammatory response attributed to physical activity. As found in the literature^39^, and in our model, there is also an increase in the amount of growth factors like HGF and EGF in the gastrocnemius. Whereas, and conversely to the literature, tissue remodelling is reduced with a decrease in soluble PECAM and in MMP2 muscle contents. Nevertheless, the right gastrocnemius mass is positively correlated with 9 markers of oxidative stress and anti-inflammatory response in the tumours. All together, these results support the hypothesis that physical activity reduces tumour growth through a modification of the tumour environment in terms of adipokine and MMPs as well as inflammatory and antioxidant status.

Finally, changes in the tumour environment resulte in crosstalk between IAT and muscles. An enriched environment is linked to a decrease in the tumour proliferative pathway ERK1/2 associated with the decrease in MMP2 and 3 as well as the tumour anti-oxidative response and COX total activity. As described in the literature, inflammation and oxidative stress are two major hallmarks of cancer and are essential with the modulation of MMP activities to sustain tumour growth and expansion^9^. Among the antioxidant response biomarkers, thioredoxin, GST, heme oxygenase and COX-2 activities decrease contrary to the tendency for the damage markers related to a high lipid per oxidation (isoprostanes) to increase in the mice housed in an EE. The positive correlations between all markers of antioxidant response, such as glutathione-s-transferase and COX-2, indicate that physical activity has a global effect on the tumour antioxidant response. Furthermore, physical activity is also linked to a decrease in intra-tumour NFκB, which is the major signalling pathway responding and controlling inflammation, as well as oxidative stress. At last, the isoprostane increase reflects cell stress^40^, confirming that physical activity plays a major role in tumour environment oxidative status.

## Conclusion

This original study allows us to highlight the changes induced by excess weight and an active daily life in mammary carcinogenesis. Our study demonstrates that, similar to intensive physical activity training, moderate spontaneous physical activity is able to counteract tumour growth. The increase of physical activity due to an enriched environment led to a decrease in inflammation and in the antioxidant response in the tumour environment. This effect does not seem to act directly on the tumour but by virtue of the surrounding tissues, through cytokine, growth factor and inflammation changes. However, large metabolites (cholesterol, triglycerides, glucose) are not altered by spontaneous physical activity, demonstrating that the hormonal signals are preponderant in tumour growth and in the activation of intracellular signalling pathway. Despite the changes observed in each tissue, moderate physical activity had no impact on plasma concentrations of inflammatory biomarkers except for adiponectin, highlighting a paracrine crosstalk between tissues. Our model confirms that one of the most important modulators in carcinogenesis is tumour oxidative stress, as previously cited in the literature^41,42^, and, further, that it could be modulated by spontaneous moderate physical activity in a paracrine manner^43^.

## Supplementary information

Supplemental tables.

## Data Availability

The datasets used and/or analysed during the current study are available from the corresponding author upon reasonable request.
