# Supplementary material for: Modulation of inter-organ signalling in obese mice by spontaneous physical activity during mammary cancer development
Source: Sci Rep. 2020 May 29;10:8794. doi: 10.1038/s41598-020-65131-9 (PMC7260359; doi:10.1038/s41598-020-65131-9)
Supplement: Supplementary file 1 — Supplemental tables. [file 41598_2020_65131_MOESM1_ESM.docx]

Modulation of inter-organ signalling in obese mice by spontaneous physical activity during mammary cancer development

Delphine Le Guennec¹, Victor Hatte¹, Marie-Chantal Farges^1^, Stéphanie Rougé^1^, Marie Goepp^1,2^, Florence Caldefie-Chezet¹, Marie- Paule Vasson^1^, Adrien Rossary^1^

1. University of Clermont Auvergne, INRA, Human Nutrition Unit, ECREIN team, BP 10448, F-63000 Clermont-Ferrand, France ;
2. University of Edinburgh, centre for regenerative medicine, Old College, South Bridge, Edinburgh EH8 9YL, Royaume-Uni.

Corresponding author: D. Le Guennec ([Delphine.le_guennec@uca.fr](mailto:Delphine.le_guennec@uca.fr))

Supplemental Table 1: High fat diet composition

| *Nutrient (g/kg)* | High fat diet |
| --- | --- |
| Casein | 230 |
| Corn starch | 200 |
| Sucrose | 220 |
| Cellulose | 80 |
| Lard | 189 |
| Soybean oil | - |
| Canola oil | 21 |
| ω6 (mg per day) | 41,9 |
| ω3 (mg per day) | 6,8 |
| *Ratio ω6/ω3* | 6,2 |
| Mineral AIN 93G-MX1 | 50 |
| Vitamin AIN 93G-VX 2 | 10 |
| Choline bitartrate | 20 |
| *Macronutrient proportion of total energy intake (%)* |  |
| Proteins | 16 |
| Carbohydrates | 39 |
| Lipids | 45 |
| Diet energy content (kcal/g) | 4.1 |

The High-fat diet was developed in collaboration with SAFE and in agreement with the AIN recommendations.

**^1^AIN 93G-MX mineral mixture (g/kg)**: calcium phosphate 500, sodium chloride 74, potassium citrate 2220, potassium sulphate 52, magnesium oxide 24, manganous carbonate 3.5, ferric citrate 6, zinc carbonate 1.6, cupric carbonate 0.3, potassium iodate 0.01, sodium selenite 0.01, chromium potassium sulphate 0.55, sucrose 118.03.

**^2^AIN 93G-VX vitamin mixture (g/kg):** thiamine HCl 0.6, riboflavin 0.6, pyridoxine HCl 0.7, niacin 3, calcium pantothenate 1.6, folic acid 0.2, biotin 0.02, vitamin B12 1, vitamin A (500,000 U/mg) 0.8, vitamin D3 (400,000 U/mg) 0.25, vitamin E acetate (500 U/g) 10, menadione sodium bisulfite 0.08, sucrose 981.15.

Supplemental table 2: Composition of groups of variables

| Tumour growth | Adipose tissues masses | | Muscles masses | | Plasma biology | Gastrocnemius biology | Inguinal adipose tissue biology | Mammary gland biology | Tumour biology | Tumour oxidative status | Environment |
| --- | --- | --- | --- | --- | --- | --- | --- | --- | --- | --- | --- |
| Days for reaching limit point | TA-visceral mass | | Leg left-mass | | IL-6 | IL-6 | IL-6 | IL-6 | IL-6 | Thiols | Standard |
| Volume | I-mass | | G right-mass | | Resistine | Resistine | Resistine | Resistine | Resistine | Glutathione reduced | Enriched |
| Weight | TA-total mass | | Leg right-mass | | Leptine | Leptine | Leptine | Leptine | Leptine | Glutathione total |  |
|  |  | |  | | Total PAI-1 | Total PAI-1 | Total PAI-1 | Total PAI-1 | Total PAI-1 | Glutathione-S-transferase |  |
|  | |  |  | Adiponectine | | Adiponectine | Adiponectine | Adiponectine | Adiponectine | Glutathione reductase |  |
|  | |  |  | PECAM-soluble | | PECAM-soluble | PECAM-soluble | PECAM-soluble | PECAM-soluble | Thioredoxine |  |
|  | |  |  |  | | Fas ligand | Fas ligand | Fas ligand | Fas ligand | COX total |  |
|  | |  |  | HGF | | HGF | HGF | HGF | HGF | COX 1 |  |
|  | |  |  | G-CSF | | G-CSF | G-CSF | G-CSF | G-CSF | COX 2 |  |
|  | |  |  | EGF | | EGF | EGF | EGF | EGF | Heme oxygenase |  |
|  | |  |  | Oestrogens | | TNF-α | TNF-α | TNF-α | TNF-α | Isoprostanes |  |
|  | |  |  | Glucose | | VEGF-α | VEGF-α | VEGF-α | VEGF-α | Glutathione peroxydase |  |
|  | |  |  | TG | | MMP-9-2-3 | MMP-9-2-3-12 | MMP-9-2-3-12 | MMP-9-2-3-12 |  |  |
|  | |  |  | Cholesterol | | CREB | CREB | CREB | CREB |  |  |
|  | |  |  |  | | NFkB | NFkB | NFkB | NFkB |  |  |
|  | |  |  |  | | JNK | JNK | JNK | JNK |  |  |
|  | |  |  |  | | P38 | P38 | P38 | P38 |  |  |
|  | |  |  |  | | ERK1/2 | ERK1/2 | ERK1/2 | ERK1/2 |  |  |
|  | |  |  |  | | AKT | AKT | AKT | AKT |  |  |
|  | |  |  |  | | P70S6K1 | P70S6K1 | P70S6K1 | P70S6K1 |  |  |
|  | |  |  |  | | STAT3 | STAT3 | STAT3 | STAT3 |  |  |
|  | |  |  |  | | STAT5 | STAT5 | STAT5 | STAT5 |  |  |
